# Effect of spironolactone on monocyte subsets in atrial fibrillation: IMPRESS-AF randomised controlled trial

**DOI:** 10.1038/s41598-024-74592-1

**Published:** 2025-07-28

**Authors:** Farhan Shahid, Eduard Shantsila, Alena Shantsila, Gregory Y H Lip

**Affiliations:** 1 Institute of Cardiovascular Sciences, University of Birmingham, Birmingham, UK; 2 Departments of Primary care and Mental Health, University of Liverpool, Liverpool, UK; 3 Liverpool Centre for Cardiovascular Science, University of Liverpool, Liverpool, UK

**Keywords:** Monocyte subsets, Atrial fibrillation, Spironolactone, Predictive markers, Biomarkers, Outcomes research

## Abstract

**Background:**

Monocyte subsets differentially influence pathophysiology of heart failure and atrial fibrillation (AF) through inflammation, fibrosis, and angiogenesis. Spironolactone has antifibrotic properties. This study investigated the effect spironolactone on monocyte subsets and monocyte effects for peak oxygen consumption (peakVO2), diastolic function and brain natriuretic peptide (BNP) in the IMPRESS-AF randomised controlled trial population (2-year treatment with spironolactone 25 mg vs placebo).

**Methods:**

CD14++CD16-CCR2+(Mon1), CD14++CD16+CCR2+(Mon2) and CD14+CD16++CCR2-(Mon3) monocyte subsets were analysed by flow cytometry and compared between spironolactone and placebo groups at 12 months and 24 months after randomisation. PeakVO2, diastolic function (echocardiographic E/'e) and BNP were measured at baseline and 24 months. Linear regression was used to assess the effects of monocytes on the outcomes (Python 3.10 modules).

**Results:**

Monocyte data were available in 225 (90%) IMPRESS-AF patients (age 72[67-77], 78% male). At 12-month the spironolactone group had fewer Mon3 (50[36-74] vs 60[44-90] cells/microL, *p*=0.02), and lower CD14 expression on Mon1 (1.37[1.17-1.59] vs 1.48[1.24-1.70], *p*=0.04); no difference remained by 24 months (*p*>0.05). A high 12-month Mon1 count independently predicted lower E/e’ at 24 months (*p*=0.02).

**Conclusions:**

Spironolactone temporarily reduced proinflammatory monocyte markers (Mon3 count and CD14 expression on Mon1). Mon1 may have a positive effect on diastolic dysfunction in AF.

## Introduction

Monocytes play important role in inflammation and have functional characteristics that may be both detrimental and beneficial to the cardiovascular system, including phagocytosis, cytokine production, collagen synthesis and angiogenesis^1^. Their functional diversity reflects the presence of distinct monocyte subsets. The mechanisms leading to reduced exercise capacity, related morbidity, and mortality in anticoagulated patients with permanent AF are related to an impairment in diastolic function, myocardial fibrosis and stiffening. Biomarkers of cardiac fibrosis have been shown to be elevated in patients with permanent AF^2^. The subsequent changes can lead to ventricular filling abnormalities, reduced cardiac output and decreasing exercise capacity^3^.

Aldosterone is an important promoter of LV fibrosis^4^. Mechanisms of aldosterone-mediated cardiac fibrosis include myocardial inflammation, oxidative stress, and cardiomyocyte apoptosis and also direct stimulation of cardiac fibroblasts to produce collagen^5,6^. In the RALES trial, the improved survival in patients systolic heart failure treated by spironolactone was linked to its ability to reduce serum markers of ongoing fibrosis (type I and III collagen synthesis)^7^.

Monocyte could facilitate fibrosis, diastolic dysfunction and related reduction in exercise tolerance and heart failure through different mechanisms: Transforming growth factor β1 production by monocytes and monocyte-derived myofibroblats^8^; release of matrix metalloproteases; into fibroblasts and promotion of chronic proinflammatory state^9^. Inhibition of monocyte accumulation suppressed myocardial fibrosis^10^. Aldosterone leads to cardiac invasion by proinflammatory mononuclear cells^11^. Multiple lines of evidence from animal models suggest the role of aldosterone and mineralocorticoid receptors in regulating monocyte function, tissue migration and differentiation in ‘inflammatory’ macrophages and their implication in cardiovascular pathogenesis. However, human data are scarce^12^.

The IMPRESS-AF randomised controlled trial (*n* = 250) compared 2-year treatment with spironolactone 25 mg daily vs. placebo for the primary outcome of peak oxygen consumption (peakVO2) on cardiopulmonary exercise testing in patients with permanent atrial fibrillation (AF) with preserved ejection fraction^13,14^. This study investigated the effect of the trial treatment on monocyte subsets and the subset effects for peakVO2, diastolic function and brain natriuretic peptide (BNP) levels in the IMPRESS-AF population.

Using the IMPRESS-AF data, the study the hypothesis that spironolactone reduces monocyte counts and monocyte expression of receptors involved in inflammation and that these monocyte characteristics are associated with exercise tolerance, diastolic dysfunction and BNP levels in patients with AF.

## Methods

### Population

Eligible participants of the IMPRESS-AF trial were people aged ≥ 50 years who had permanent AF, echocardiographic LVEF ≥ 55% at recruitment^13,14^. Exclusion criteria included: Life expectancy of less than two years, left ventricular ejection fraction < 50% on echocardiography, severe lung disease, severe valvular heart disease, serum creatinine > 220 µmol/L, potassium > 5 at baseline screening, recent coronary artery bypass (within last 3 months), use of potassium sparring diuretic or aldosterone antagonist within previous 14 days, systolic blood pressure < 160mmHg, and be able to perform cardiopulmonary exercise testing using a cycling ergometer. BNP values were measured at baseline and 24 months but were not part of the eligibility criteria.

A total of 250 patients were recruited from primary care and outpatient AF clinics in Sandwell and West Birmingham Hospitals Trust, Birmingham. All patients were screened, investigated, and managed in the Research Clinic at the Institute of Cardiovascular Sciences, City Hospital, Birmingham. The study was approved by the National Research and Ethics Committee West Midlands—Coventry and Warwickshire (REC Reference 14/WM/1211). All patients provided signed informed consent. All methods were performed in accordance with the relevant guidelines and regulations.

### Study treatment

Participants were randomised 1:1 to receive spironolactone 25 mg daily or placebo for 2 years in double blind manner. The study is registered at EudraCT number 2014- 003702-33 (date of first trial registration 23/01/2015) and Clinicaltrial.gov (NCT02673463, date of first trial registration 04/02/2016).

### Cardio-pulmonary exercise testing (CPET)

CPET testing with oximetry was performed to assess PeakVO2 (COSMED Srl, Rome, Italy)^15–17^. Exercise testing will be performed with patients in the upright position on an electronically braked bicycle, with expired gas analysis under continuous electrocardiographic and blood pressure monitoring^18^. Participants were encouraged to exercise to exhaustion. Peak VO2 values were averaged from the final 30 s of the exercise test. CPET was done at baseline and at 24 months.

#### Echocardiography

Transthoracic echocardiography was performed using Phillips iE33 ultrasound system (Bothel, WA, USA) at baseline and after 24 months. Average values from 10 consecutive cardiac cycles were calculated to establish the LVEF and the ratio of mitral peak velocity of early filling (E) to early diastolic mitral annular velocity (E′) (E/E′ ratio).

#### Flow cytometry

Following a well-established and validated protocol, the monocyte subsets were analysed using a BD FACS Calibur flow cytometer. Briefly, mouse anti-human monoclonal fluorochrome-conjugated antibodies anti-CD16-Alexa Fluor 488 (clone DJ130c; AbD Serotec, Oxford, UK), anti-CD14-PE (clone MφP9; BD), anti-CD42a-PerCP (clone Beb1; BD) and anti-CCR2-APC (clone 48607, R&D Systems, Abingdon, UK) were mixed with 50 µl of fresh EDTA anticoagulated whole blood in TruCount tubes (BD, Oxford, UK) containing a strictly defined number of fluorescent count beads, followed by blood lysis and immediate acquisition by flow cytometer. Monocyte subsets were defined as CD14 + + CD16 − CCR2+ (Mon1, classical), CD14 + + CD16 + CCR2+ (Mon2, intermediate) and CD14 + CD16 + + CCR2− (Mon3, non-classical) in accordance with contemporary nomenclature^19^. The technique is robust and highly reproducible. The laboratory coefficient of variation for absolute monocyte count is 1.9%, and for surface, markers is < 5%^20–22^. CD14 and CD16 positivity were defined using appropriate isotype controls and in keeping with current consensus guidance. The following monocyte data were analysed for each subset: total count, count of monocyte-platelet aggregates (MPA, defined as monocyte bearing the platelet marker, CD42a), surface expression of key monocyte markers related to immune/inflammatory responses, CD14 and CD16, CCR2 (receptor for monocyte chemotactic protein-1, MCP-1).

Monocyte data were analysed twice, 12 months and 24 months since randomisation. Baseline monocyte data were not collected, considering both groups will have equal values after randomisation.

### Statistical analysis

The demographic data and clinical characteristics were presented as a median [interquartile range, IQR] where appropriate. Categorical data are presented as percentages. Comparisons between spironolactone and placebo groups were made using Mann-Whitney. Linear regression (univariate and adjusted for age, sex and spironolactone) was used to assess the effects of monocytes measured at 12 months on the study outcomes (PeakVO2, E/e’, and BNP at 24 months). Two-sided *p*-values of < 0.05 were considered statistically significant. Statistical analyses were done using Python 3.10 (Pandas, Numpy, Scipy and Statsmodels libraries).

## Results

Monocyte data were available in 225 patients (90% for the IMPRESS-AF population, 115 in placebo groups, 110 in the spironolactone group, median [interquartile range] age 72 [67–77] years, 78% male). There were no differences in baseline demographic and clinical characteristics of the participants, indicative of alignment with the random treatment allocation principle of the trial (Table 1). Monocyte data at 24 months were available for 205 patients (105 in placebo groups, 100 in the spironolactone group).

**Table 1:** Baseline demographic and clinical characteristics.

	Placebo	Spironolactone	*P*
**Baseline**	*n* = 115	*n* = 110	
Age, years	72 [67–77]	72 [68–77]	0.99
Male sex, n [%]	88 [77%]	88 [80%]	0.63
Smoking, n [%]			0.69
*current smoker*	7 [6%]	4 [4%]	
*ex-smoker*	61 [53%]	59 [54%]	
*never smoked*	47 [41%]	47 [43%]	
White ethnicity, n [%]	108 [94%]	103 [94%]	1.00
Diabetes, n [%]	21 [18%]	23 [21%]	0.74
BMI, kg/m^2^	30 [26–33]	29 [26–33]	0.94
Systolic BP, mmHg	129 [118–142]	130 [118–140]	0.89
Diastolic BP, mmHg	74 [67–82]	75 [68–82]	0.61
Heart rate, bpm	82 [74–97]	84 [74–97]	0.81
BNP, pg/mL	126 [81–215]	116 [73–205]	0.43
PeakVO_2_, mL/kg/min	14.5 [10.9–18.6]	14.2 [11.0-18.6]	0.93
LVEF, %	58 [56–64]	58 [56–62]	0.51
E/e’	9.6 [7.4–12.8]	9.8 [8.0-11.9]	0.90
eGFR, mL/min/1.73m^2^	70 [59–82]	72 [61–85]	0.51
Total cholesterol, mmol/L	4.1 [3.5–4.9]	4.1 [3.6–4.8]	0.91
LDL cholesterol, mmol/L	2.1 [1.7–2.9]	2.1 [1.5–2.8]	0.39
HDL cholesterol, mmol/L	1.3 [1.1–1.5]	1.3 [1.1–1.5]	0.75
**24-month follow**	*n* = 105	*n* = 100	
BMI, kg/m2	29 [27–34]	30 [26–34]	0.76
Systolic BP, mmHg	129 [118–141]	121 [112–134]	**0.002**
Diastolic BP, mmHg	73 [67–82]	71 [65–78]	0.14
Heart rate, bpm	78 [69–93]	80 [71–92]	0.55
BNP, pg/mL	167 [105–243]	141 [94–205]	0.13
PeakVO_2_, mL/kg/min	14.7 [11.4–18.0]	14.0 [11.6–18.1]	0.83
LVEF, %	57 [55–60]	58 [55–60]	0.78
E/e’	9.4 [7.2–11.2]	8.6 [6.8–10.4]	0.09

Data presented as median [interquartile range].

BNP, brain natriuretic peptide; BMI, body mass index; BP, blood pressure; bpm, beats per minute; eGFR, estimated glomerular filtration rate; HDL, high density lipoproteins; LDL, low density lipoproteins; LVEF, left ventricular ejection fraction; PeakVO2, peak oxygen consumption on cardio-pulmonary exercise texting.

At 12-month the spironolactone group had fewer Mon3 (50 [36–74] vs. 60 [44–90] cells/microL, *p* = 0.02), and monocyte platelet aggregates with Mon3 (6.0 [3.4–9.5] vs. 7.7 [4.8–11.2] cells/microL, *p* = 0.01) and lower expression of CD14 on Mon1 (1.37 [1.17–1.59] vs. 1.48 [1.24–1.70], *p* = 0.04), but no significant difference remained by 24 months (*p* > 0.05 for all monocyte characteristics) (Table 2).

**Table 2:** Monocyte characteristics.

	12 months			24 months		
	Placebo *n* = 115	Spironolactone *n* = 110	*p*	Placebo *n* = 105	Spironolactone *n* = 100	*p*
*Monocyte counts*,* per microL*						
Total monocytes	671 [545–823]	649 [511–793]	0.17	813 [632–1014]	790 [588–923]	0.46
Mon1	550 [437–667]	531 [412–639]	0.21	676 [534 − 507]	678 [507–827]	0.36
Mon2	41 [24–65]	43 [25–79]	0.50	24 [13–51]	23 [11–46]	0.81
Mon3	60 [44–90]	50 [36–74]	**0.02**	67 [50–93]	69 [52–93]	0.94
MPA total	58 [42–79]	55 [35–77]	0.31	68 [52–88]	67 [53–93]	0.96
MPA with Mon1	38 [27–53]	37 [23–52]	0.44	53 [37–68]	50 [34–72]	0.86
MPA with Mon2	7.0 [4.6–11.8]	7.3 [4.4–10.6]	0.72	7.5 [5.1–10.2]	7.1 [4.7–11.2]	0.75
MPA with Mon3	7.7 [4.8–11.2]	6.0 [3.4–9.5]	**0.01**	8.5 [6.8–12.6]	9.4 [6.7–14.1]	0.34
*Surface expression*,* median fluorescent intensity*						
CD14 Mon1, x10^3^	1.48 [1.24–1.70]	1.37 [1.17–1.59]	**0.04**	1.12 [1.00-1.31]	1.10 [0.91–1.31]	0.32
CD14 Mon2, x10^3^	1.29 [0.93–1.51	1.29 [0.92–1.55]	0.93	0.69 [0.39–1.01]	0.67 [0.42–0.95]	0.92
CD14 Mon3, x10^3^	0.18 [0.15–2.26]	0.19 [0.15–0.23]	0.61	136 [107–170]	133 [114–162]	0.70
CD16 Mon1	18.3 [15.5–21.3]	17.8 [14.8–21.9]	0.82	11.9 [10.1–14.2]	12.1 [10.9–15.0]	0.30
CD16 Mon2	112 [98–129]	107 [96–121]	0.26	126 [105–166]	123 [106–169]	0.66
CD16 Mon3	236 [177–292]	242 [196–310]	0.29	151 [109–210]	149 [114–199]	0.85
CCR2 Mon1	150 [92–214]	159 [98–207]	0.54	100 [75–136]	96 [71–139]	0.49
CCR2 Mon2	116 [90–149]	126 [92–162]	0.28	93 [79–114]	86 [74–110]	0.19
CCR2 Mon3	13.8 [12.2–15.6]	14.3 [12.5–15.5]	0.35	13.5 [11.9–14.8]	13.3 [12.1–15.1]	0.72

Data presented as median [interquartile range].

On univariate linear regression, the following 12-month monocyte markers were significantly associated with the study outcomes (Table 3): CD16 expression on Mon3 (*p* = 0.001) for PeakVO2; CD16 expression on Mon3 (*p* < 0.001) and CCR2 expression on Mon2 (*p* = 0.04) for BNP; counts Mon1 (*p* = 0.04) and MPA associated with Mon1 (*p* = 0.045). A high 12-month Mon1 count independently predicted lower E/e’ at 24 months (regression coefficient − 0.003, standard error 0.001, *p* = 0.022); higher CCR2 expression on Mon2 was independently associated with lower BNP (regression coefficient − 0.21, standard error 0.10, *p* = 0.038). Spironolactone was not significantly associated with the study outcome (*p* > 0.05 for all) (Table 4).

**Table 3:** Predictive value of 12-month monocyte characteristic for end of study outcomes.

Marker	Mon1		Mon2		Mon3		
	B±SE	*p*	B±SE	*p*	B±SE		*p*
*Outcome: PeakVO*_*2*_							
Subset count	0.0004 ± 0.002	0.82	0.003 ± 0.005	0.60	-0.0009 ± 0.003		0.72
MPA count	-0.01 ± 0.01	0.44	-0.04 ± 0.04	0.32	-0.03 ± 0.03		0.22
CD14^*^	-0.0003 ± < 0.001	0.39	0.0004 ± 0.001	0.53	-0.003 ± 0.002		0.16
CD16^*^	-0.02 ± 0.07	0.74	0.04 ± 0.01	0.54	0.01 ± 0.003		0.001
CCR2^*^	0.005 ± 0.004	0.23	0.004 ± 0.01	0.50	0.09 ± 0.12		0.47
*Outcome: BNP*							
Subset count	0.04 ± 0.05	0.38	0.04 ± 0.17	0.81	-0.05 ± 0.08		0.51
MPA count	-0.15 ± 0.42	0.71	-0.26 ± 1.33	0.87	0.65 ± 0.83		0.43
CD14^*^	-0.01 ± 0.01	0.30	-0.03 ± 0.02	0.21	0.08 ± 0.07		0.24
CD16^*^	-0.68 ± 2.21	0.76	0.12 ± 0.23	0.61	-0.34 ± 0.10		< 0.001
CCR2^*^	-0.21 ± 0.12	0.09	-0.41 ± 0.20	0.04	-4.49 ± 3.69		0.23
*Outcome: E/e’*							
Subset count	-0.002 ± 0.04	0.04	-0.005 ± 0.004	0.20	0.002 ± 0.002		0.34
MPA count	-0.02 ± 0.01	0.045	-0.04 ± 0.03	0.16	0.01 ± 0.02		0.52
CD14^*^	-0.0001 ± < 0.001	0.54	-0.0002 ± < 0.001	0.70	-0.002 ± 0.002		0.25
CD16^*^	-0.05 ± 0.05	0.37	0.005 ± 0.005	0.92	-0.003 ± 0.002		0.17
CCR2^*^	< 0.001 ± 0.003	0.99	-0.002 ± 0.005	0.70	-0.12 ± 0.09		0.18

Linear regression analysis.

^*^Surface marker expression.

B, regression coefficient; BNP, brain natriuretic peptide; MPA, monocyte-platelet aggregates; PeakVO2, peak oxygen consumption on cardio-pulmonary exercise texting; SE, standard error.

**Table 4:** Predictive value of 12-month monocyte characteristics for end of study outcomes.

Monocyte marker			Age		Sex		Spironolactone	
	B ± SE	*p*	B ± SE	*p*	B ± SE	*p*	B ± SE	*p*
*Outcome: PeakVO2*								
Mon3 CD16^*^	0.004 ± 0.003	0.24	-0.16 ± 0.04	< 0.001	3.02 ± 0.73	< 0.001	-0.36 ± 0.58	0.54
*Outcome: BNP*								
Mon3 CD16^*^	-0.21 ± 0.10	0.038	4.21 ± 1.40	0.03	-40.3 ± 23.7	0.09	1.76 ± 19.0	0.93
Mon2 CCR2^*^	-0.30 ± 0.19	0.12	4.89 ± 1.36	< 0.001	-45.4 ± 23.6	0.06	0.88 ± 19.0	0.96
*Outcome: E/e’*								
Mon1 count	-0.003 ± 0.001	0.022	0.10 ± 0.03	0.003	-0.17 ± 0.57	0.77	-0.88 ± 0.45	0.053
Mon1 MPA count	-0.02 ± 0.01	0.06	0.09 ± 0.03	0.006	-0.31 ± 0.56	0.58	-0.79 ± 0.45	0.08

Linear regression analysis.

^*^Surface marker expression.

B, regression coefficient; BNP, brain natriuretic peptide; MPA, monocyte-platelet aggregates; PeakVO2, peak oxygen consumption on cardio-pulmonary exercise texting; SE, standard error.

## Discussion

This study has shown for the first time that spironolactone, compared to placebo, temporarily changes monocyte subsets at 12 months, but the difference no longer exists at 24 months. The 12-month changes are consistent with anti-inflammatory treatments in monocyte patterns (lower Mon3 counts and their aggregates with platelets and lower CD14 expression on Mon1). In the IMPRESS-AF, spironolactone reduced systolic BP but did not influence PeakVO2, E/E’, BNP, and LVEF^13,14^. BP is not known to influence monocytes, so it is likely that either spironolactone directly changes monocyte function or indirectly influences it by inhibiting mineralocorticoid receptors in other tissues. The latter possibility appears more likely as monocyte life in circulation is relatively short, and the lack of monocyte changes at 24 months indicates tissue adjustment to spironolactone effects.

Previous data showed that healthy volunteers and patients with stable cardiac disease have similar counts and phenotypic characteristics of monocyte subsets (the typical split of monocyte numbers is about 85% Mon1, 6% Mon2, and 9% Mon3)^20–24^. Despite this, in patients with AF, higher monocyte counts have a strong independent predictive value for the risk of major adverse cardiovascular events, major bleeding and death^25^. The study provides in-human evidence of monocyte effects of aldosterone inhibition, which complements data from in vitro and animal models. Aldosterone stimulates mineralocorticoid receptor-dependent induction of the vascular cell adhesion molecule-1 (VCAM-1) and intercellular adhesion molecule (ICAM-1) in endothelial cells, promoting monocyte migration into the vascular wall^26–29^. Mice models of hypertension indicated that mineralocorticoid receptor activation facilitates a pro-inflammatory shift, with increased circulating interleukin 6 and MCP-1 levels^30^. Upon activation, monocytes differentiate into macrophages with diverse phenotypes and other cell types defining their tissue functions. Scarce data, based on a rat model, indicate aldosterone infusion led to renal macrophage accumulation and skewed macrophages phenotype towards the ‘inflammatory’ M1 phenotype^31^. Stimulation of the mineralocorticoid receptor increases the production of reactive oxygen species, metalloproteinases and consequently, fibrosis^32,33^.

Admittedly, the role of Mon3 in humans remains enigmatic. Its counts are not affected by acute cardiovascular pathology and human monocyte subsets^21,22^. Our study indicates that Mon3 may be more involved in chronic processes, supported by the notion of an independent association of its phenotype (CD16, the defining marker of the subset) with BNP levels and suppression of Mon3 count by 12-month treatment with spironolactone (may either reflect lower production in bone marrow or accelerated migration to tissues). The magnitude of the effects appears relatively small, and it is likely a contributing rather than a major aetiological factor of heart failure with preserved ejection fraction in AF.

Such observational associations are hypothesis-generating and align with previous suggestions (albeit not shown before in this study setting) that there is a potential role for Mon3 and its key receptor CD16 in ‘tissue repair’^34^. With Mon3 shown to have a role in limiting adverse remodelling in patients with cardiovascular disease, one can hypothesise that the process of adverse left atrial remodelling can be augmented depending on the differentiation of monocyte subsets in the bone marrow^35^. One may hypothesise that monocyte subsets play a key role in the process of inflammation and subsequent fibrosis that has been shown in non-AF patients with cardiovascular disease^21,36^.

The findings regarding Mon1 are also of interest. Mon1 expression of CD14 (lipopolysaccharide receptor) implicated in inflammatory responses of this subset was reduced by 12-month spironolactone treatment. However, lower subsets counts were independently associated with worse diastolic function. The observation mechanisms are unclear and likely related to the still poorly understood cytokine and chemokine microenvironment created by the subsets after migration to tissues.

## Limitations

Despite the advantages of being based on a randomised clinical trial, the study has several limitations. They did not analyse the baseline monocyte subsets, and the magnitude of spironolactone effects is based on using a placebo as a reference group. Detailed analysis of the mechanistic effects of spironolactone on monocytes and monocyte myocardial effects are beyond the scope of the study, markers of systemic inflammation have not been accounted for and there are still major methodological limitations tracking monocyte fate and function after migration to tissues. The study mainly included white participants, and generalisability to other ethnic groups is unclear.

## Conclusions

In a randomised controlled trial, spironolactone reduced proinflammatory monocyte markers (Mon3 count and CD14 expression on Mon1) at 12 months of treatment, but the difference was transient and lost by 24 months. Mon1 may have a positive effect on diastolic dysfunction in AF.

## Data Availability

The data that support the findings of this study are available from the University of Birmingham, but restrictions apply to the availability of these data, which were used under license for the current study, and so are not publicly available. Data are however available from the authors upon reasonable request and with permission of University of Birmingham (please contact Eduard Shantsila, the corresponding author).
